# Restrictive lung disorder is common in patients with kidney failure and associates with protein-energy wasting, inflammation and cardiovascular disease

**DOI:** 10.1371/journal.pone.0195585

**Published:** 2018-04-27

**Authors:** Hideyuki Mukai, Pei Ming, Bengt Lindholm, Olof Heimbürger, Peter Barany, Björn Anderstam, Peter Stenvinkel, Abdul Rashid Qureshi

**Affiliations:** 1 Division of Renal Medicine and Baxter Novum, Department of Clinical Science, Intervention and Technology, Karolinska Institutet, Stockholm, Sweden; 2 Renal Department, First affiliated Teaching Hospital, Tianjin University of Traditional Chinese Medicine, Tianjin, China; Kaohsiung Medical University Hospital, TAIWAN

## Abstract

**Background:**

Cardiovascular disease (CVD), protein-energy wasting (PEW), and inflammation are common interrelated features of chronic kidney disease (CKD). Less is known about lung dysfunction in CKD and its possible role in this context. We evaluated lung function and its association with estimated glomerular filtration rate (GFR), CVD, PEW, and inflammation in individuals with normal to severely reduced GFR.

**Methods:**

In 404 individuals with GFR category G1 (n = 31; GFR >90mL/min/1.73 m^2^), G2 (n = 46), G3 (n = 33), G4 (n = 49) and G5 (n = 245; GFR<15mL/min/1.73 m^2^), pulmonary function was assessed by spirometry. Obstructive (OLD) and restrictive (RLD) lung dysfunction was defined based on forced vital capacity (FVC), forced expiratory volume in the first second (FEV_1_) and peak expiratory flow (PEF), expressed as percentages of predicted values (%FEV_1_, %FVC and %PEF, respectively). PEW was evaluated by subjective global assessment, handgrip strength (HGS) and lean body mass index (LBMI), and inflammation by interleukin-6 and high sensitivity C-reactive protein.

**Results:**

RLD (defined as FEV_1_/FVC ≥ 0.70 and %FVC<80) associated with GFR and was present in 36% of G5 and 14% of G1-4 individuals. OLD (FEV_1_/FVC<0.70) was less common with similar prevalence among G1-4 (9%) and G5 (11%) individuals. Notably, 64% of those with concomitant presence of PEW, inflammation and clinical signs of CVD had RLD while 79% of those lacking these complications had normal lung function. In multivariate logistic regression analysis, RLD associated with CVD, PEW and inflammation, after adjusting for Framingham’s CVD risk score, serum albumin, and GFR category.

**Conclusions:**

RLD is a common complication in patients with advanced CKD, especially in those with concomitant presence of CVD, inflammation and PEW. RLD appears to be an integral albeit scarcely explored consequence of pulmonary-renal interactions in CKD patients.

## Introduction

Non-infectious pulmonary complications are common in patients with advanced chronic kidney disease (CKD) [1], especially in those with volume overload, and include pulmonary edema, pleural effusions, increased pulmonary capillary permeability, pulmonary fibrosis, pulmonary calcification, respiratory muscle myopathy and low respiratory muscle strength [2, 3]. As a consequence, lung function is often impaired with spirometry showing low forced vital capacity (FVC), low forced expiratory volume in the first second (FEV1), and low peak expiratory flow (PEF) indicating *obstructive impairment* (defined as FEV_1_/FVC < 0.70) or *restrictive impairment* (defined as FEV_1_/FVC ≥ 0.70, and %FVC < 80) [4]. Patients with impaired lung function are at high risk of death not only from pulmonary but also from cardiovascular disease, CVD [5].

Inflammation, protein-energy wasting (PEW) and CVD are common interlinked features of the uremic phenotype [6, 7]. Inflammation is inherently associated with uremia due to immune cell dysfunction [8] leading to increased susceptibility to infections [9]. In CKD, an inflammatory state [10, 11] is sustained by impaired renal elimination of pro-inflammatory cytokines and increased generation of cytokines in the uremic milieu [12]. A low glomerular filtration rate (GFR) associates with elevated circulating concentrations of pro-inflammatory cytokines, such as interleukin-6 (IL-6) and tumor necrosis factor (TNF), that may induce muscle proteolysis, increase energy expenditure and decrease appetite, thereby contributing to PEW [13, 14].

Inflammation has been linked to impaired lung function in the general population [5, 15, 16] and in patients with chronic obstructive pulmonary disease (COPD) to decreased respiratory muscle mass and increased mortality risk [17]. Low muscle mass and strength associate with increased mortality in CKD patients [18–20]. In patients with advanced CKD, PEW is common [7, 21, 22] and associated with cardiovascular morbimortality [23, 24]. Loss of muscle mass and strength induced by inflammation and PEW may affect respiratory muscles and could contribute to impaired pulmonary function in CKD stage 5 patients [25, 26]. However, the prevalence, characteristics, and prognostic implications of pulmonary dysfunction among patients with earlier stages of CKD are less well known.

We analyzed in a cross-sectional study the prevalence and characteristics of lung dysfunction, and its association with CVD, inflammation and PEW, across different GFR categories. The results suggest that inflammation and PEW are key factors in pulmonary-cardio-renal interactions resulting in high prevalence of restrictive lung disorder in patients with advanced CKD.

## Materials and methods

### Individuals and study design

Pulmonary function was investigated by spirometry in 404 clinically stable individuals with GFR values ranging from normal levels to levels corresponding to kidney failure. Exclusion criteria were age <18 years, acute renal failure, signs of overt clinical infection and unwillingness to participate. Informed consent was obtained from each individual and the Ethics Committee of the Karolinska Institutet at Huddinge University Hospital approved the study. The study was conducted in adherence to the Declaration of Helsinki.

GFR categories G1-5 were defined according to current recommendations [27] based on estimated GFR calculated by the CKD-EPI (CKD Epidemiology Collaboration) equation [28] according to the National Kidney Foundation’s K/DOQI guidelines [29]. We included individuals with normal (G1; n = 31; GFR >90mL/min/1.73 m^2^) or only mildly decreased (G2; n = 46) GFR, and CKD patients with significant reductions of GFR, G3 (n = 33) and G4 (n = 49), as well as patients with kidney failure, G5 (n = 245; GFR<15mL/min/1.73 m^2^). Sources of recruitment and underlying causes of CKD were as follows:

**G1** (n = 31) and **G2** (n = 46) individuals were recruited from a population-based sample of individuals from the Stockholm region, randomly selected by Statistics Sweden (a government agency).

**G3** individuals (n = 33) included CKD patients recruited from the PRIMA study [30] (n = 31) and control subjects in the PRIMA study who had signs of CKD (n = 2). The causes of CKD were chronic glomerulonephritis (n = 6), diabetic nephropathy (n = 4), others (n = 21) and unknown (n = 2).

**G4** individuals (n = 49) included CKD patients recruited from an ongoing study of associations between malnutrition, inflammation and atherosclerosis in CKD patients initiating dialysis therapy, the MIA study [7] (n = 6), and from the PRIMA study [30] (n = 43). The causes of CKD were chronic glomerulonephritis (n = 12), hypertension and reno-vascular disease (n = 2), diabetic nephropathy (n = 8) and others (n = 27).

**G5** individuals (n = 245) included CKD patients with kidney failure not yet on dialysis who were recruited from the MIA study [7] (n = 224), and from the PRIMA study [30] (n = 21). The causes of renal failure were chronic glomerulonephritis (n = 54), hypertension and reno-vascular disease (n = 55), diabetic nephropathy (n = 74) and others (n = 62).

All individuals underwent anthropometric, biochemical and clinical assessments on the same day or close to the investigation of lung function. Their clinical and laboratory characteristics including GFR levels are shown in S1 Table.

### Pulmonary function

Spirometry assessments of forced vital capacity (FVC), forced expiratory volume in the first second (FEV_1_) and peak expiratory flow (PEF) were obtained using Spirolab (Medical International Research, Rome, Italy) with flow accuracy ±5% and volume accuracy ±3%. Predicted normal values were calculated using the formulas by Crapo et al [31] and expressed as %FEV_1_, %FVC and %PEF. At least three reproducible tests were carried out for each measurement and the highest was recorded. Pulse oximetry yielding oxygen saturation (SpO_2_) was performed with a Datex-Engström Finger Sensor (Datex-Engström Division, Instrumentarium Division, Finland) measuring red and infrared light absorption with accuracy ±2% in the SpO_2_ interval 80–100%. Obstructive lung disorder (OLD) was defined as FEV_1_/FVC < 0.70, and restrictive lung disorder (RLD) as the FEV_1_/FVC ≥ 0.70, and %FVC < 80 [4]. Based on ICD-10 codes, pre-existing pulmonary disease documented in medical records was present among 24 out of 404 CKD individuals at baseline: upper respiratory infection (n = 3); pneumonia (n = 5); bronchitis (n = 2); chronic obstructive pulmonary disease, COPD (n = 10); asthma (n = 2); interstitial pulmonary disease (n = 1) and pneumothorax (n = 1). None of these 24 patients exhibited signs of overt clinical infection at the time of investigation.

### Biochemical analysis

Blood samples were collected at baseline evaluation after overnight fasting. The plasma was separated within 30 minutes, and samples were kept frozen at –70° C if not analyzed immediately. Analyses of plasma high-sensitivity C-reactive protein (hsCRP; coefficient of variation, CV, 5%), cholesterol, triglycerides, HDL-cholesterol, calcium, phosphate, hemoglobin, creatinine, and albumin (CV 3–4%) were performed at the Clinical Laboratory of Karolinska University Hospital, Stockholm, Sweden. Interleukin-6, IL-6 (CV 4%) and insulin-like growth factor-1, IGF-1 (CV 4.3%) and tumor necrosis factor (TNF) were measured on an Immulite 1000, Automatic Analyzer (Siemens Healthcare, Diagnostics Products Ltd, Los Angeles, CA, USA) at the laboratory of Department of Renal Medicine using assays manufactured for this analyzer and according to the manufacturer’s instructions. Urine albumin excretion was calculated from collection of 24-h urine sample, and those with urine albumin excretion ≥ 30mg/day were labelled as having albuminuria. Presence of CVD was defined as a clinical history or signs of ischemic cardiac disease, and/or presence of peripheral vascular disease, cerebrovascular disease, heart failure and arrhythmia. Blood pressure is reported as mean blood pressure defined as [diastolic pressure + (systolic pressure–diastolic pressure) / 3].

### Nutritional assessment

According to the subjective global assessment (SGA) score, the patients were classified as well nourished (SGA = 1) or as having mild (SGA = 2), moderate (SGA = 3) or severe (SGA = 4) malnutrition [32], and for simplicity, placed into two groups; malnourished (SGA≥2; indicating PEW) and well nourished (SGA = 1). Handgrip strength (HGS) was evaluated in the non-dominant arm using the Harpenden dynamometer (Yamar, Jackson, MI, USA) and repeated three times, and the greatest value was recorded and expressed as kg. The individual values for HGS were expressed as % of control subjects (G1 and G2, see above), adjusting for the gender, when included in the statistical analyses. Body mass index (BMI) was calculated as weight in kilograms divided by the square of height in meters. Lean body mass and fat mass were calculated by anthropometry with measurements of biceps, triceps, subscapular and supra-iliac skinfold thickness using the Durnin and Womerslesy caliper method [33], and by equations proposed by Siri [34]. Lean body mass index (LBMI) and fat body mass index (FBMI) were calculated according to the method of Kyle et al [35] and expressed as kg/m^2^. Physical activity was estimated by questionnaire including four domains: 1) exercise frequently, 2) normal activity, 3) low activity, and 4) bedridden or wheelchair bound.

Bone mineral density (BMD) was measured by dual-energy X-ray absorptiometry (DXA) using Lunar Prodigy 10631 (GE Medical Systems, Madison, WI, USA) or DPX-L device (GE Lunar iDXA, GE Medical systems, St. Giles, UK).

### Assessment of Framingham’s CVD risk

The Framingham’s CVD risk score (FRS) was calculated according to gender- and age- stratified tables with specific scores assigned for systolic blood pressure (SBP), diabetes mellitus (DM), anti-hypertensive medication, total cholesterol, HDL-cholesterol and smoking status [36].

### Statistical analysis

Data are expressed as median (10th to 90th percentile) or percentage, as appropriate. Statistical significance was set at the level of P <0.05. Comparisons between three groups were assessed with the non-parametric Kruskal-Wallis test for continuous variables and Chi-square test for nominal variables. We used multivariate logistic regression analysis to examine factors associated with the lung dysfunction. The following factors were considered in the multivariable logistic model: Framingham’s CVD risk score presence of CVD, inflammation, SGA score, and GFR categories. Statistical analyses were performed using statistical software SAS version 9.4 (SAS Campus Drive, Cary, NC, USA) and Stata 15.1 (Stata Corporation, College Station, TX, USA).

## Results

### Baseline characteristics

Clinical and biochemical characteristics of 404 individuals with GFR categories G1-5, and classified as having obstructive (OLD; n = 42; 10%) or restrictive (RLD; n = 110; 27%) lung disorders or normal (n = 252; 63%) lung function at baseline, are presented in Table 1. The age and sex distribution were similar among the three groups. Individuals with RLD had higher prevalence of DM (46%), CVD (48%), and malnutrition (SGA>1; 31%), lower %HGS, higher concentrations of CRP and IL-6, and lower GFR. Individuals with lung dysfunction were more often prescribed β-blockers and diuretics. Prednisolone tablets or inhaler were used by 27 patients and bronchodilators by 18 patients. Three patients were reported to have congestive heart failure.

**Table 1:** Clinical and biochemical characteristics of 404 individuals with various degrees of impairment of renal function divided according to spirometry results indicating normal lung function, obstructive lung disorder (OLD) or restrictive lung disorder (RLD).

	**Normal** (n = 252)	**OLD** (n = 42)	**RLD** (n = 110)	**P value**
***Demography and clinical characteristics***				
Age (years)	57 (36–71)	63 (32–72)	59 (37–71)	0.14
Males, (%)	(63)	(67)	(68)	0.62
Diabetes mellitus, (%)	(21)	(26)	(46)	<0.001
Cardiovascular disease, (%)	(18)	(31)	(48)	<0.001
Framingham CVD risk score (%)	17.8 (3.8–56.3)	31.3 (4.5–58.3)	24.9 (5.3–61.0)	0.01
Smoker, (%)	(39)	(30)	(37)	0.56
Mean BP (mmHg)	109 (91–125)	106 (90–137)	107 (90–130)	0.99
Low physical activity ^a^, n (%) (n = 234/36/105)	14 (6)	6 (17)	24 (23)	<0.001
Urine albumin ≥ 30mg/day, n (%) (n = 190/26/65)	121 (64)	19 (73)	54 (83)	0.009
eGFR (ml/min/1.73m^2^)	13.5(4.6–88.9)	9.8(4.4–76.4)	6.9(3.8–68.1)	<0.001
***Nutritional status***				
PEW (SGA>1), n (%) (n = 250/40/109)	17 (7%)	12 (30%)	34 (31%)	<0.001
Body mass index, (kg/m^2^)	25.2 (20.2–30.8)	22.9 (18–28.6)	24.7 (20.3–32.4)	0.004
Lean body mass index, (kg/m^2^, n = 249/40/101)	17.4 (14.6–20.5)	16.3 (13.2–19.7)	17.3 (13.6–20.6)	0.03
Fat body mass index, (kg/m^2^, n = 249/40/101)	7.9 (4.6–11.7)	6.8 (3.9–10.8)	7.5 (4.1–12.6)	0.06
% HGS^b^ (%, n = 251/41/107)	100 (70–122)	80 (50–106)	85 (55–122)	<0.001
Total BMD (g/cm^2^, n = 238/37/93)	1.17 (1.02–1.31)	1.14 (0.98–1.29)	1.15 (1.03–1.33)	0.30
***Markers of metabolism and nutrition***				
Hemoglobin (g/L)	123 (94–148)	116 (86–146)	109 (93–138)	<0.001
Albumin (g/L)	37 (30–42)	35 (27–41)	34 (24–40)	<0.001
IGF-1 (μg /mL, n = 204/29/86)	155 (81–307)	170 (53–234)	158 (65–289)	0.62
Triglyceride (mmol/L, n = 249/42/109)	1.6 (0.7–3.3)	1.7 (0.9–3.3)	1.7 (0.9–3.3)	0.57
Total cholesterol (mmol/L, n = 251/42/109)	5.0 (3.6–6.6)	5.1 (3.7–7.0)	4.4 (3.1–6.6)	<0.001
HDL cholesterol (mmol/L, n = 216/39/107)	1.3 (0.9–2.1)	1.2 (0.7–2.1)	1.2 (0.8–2.0)	0.22
Calcium (mmol/L, = 251/40/108)	2.4 (2.2–2.8)	2.5 (2.1–2.7)	2.4 (2.1–2.7)	0.22
Phosphate (mmol/L, n = 250/40/108)	1.4 (0.9–2.5)	1.5 (0.9–2.6)	1.8 (0.9–2.7)	<0.001
Intact-PTH (ng/L, n = 248/42/110)	103 (30–398)	93 (30–482)	197 (33–469)	<0.001
***Biomarkers of inflammation***				
hsCRP (mg/L)	2.5 (0.5–14.0)	4.3 (0.8–52.3)	7.7 (1.1–30.8)	<0.001
IL-6 (pg/mL, n = 214/37/105)	3.3 (1.2–10.1)	8.1 (2.2–18.7)	6.3 (2.3–21.7)	<0.001
Fibrinogen (g/L, n = 247/41/108)	3.7 (2.6–5.9)	4.1 (2.8–6.4)	5.0 (3.1–6.9)	<0.001
TNF (pg/mL, n = 194/35/96)	8.9 (4.1–16.7)	10.3 (3.9–27.4)	12.8 (6.5–21.4)	<0.001
Leucocytes count (10^9^/L)	6.8 (4.7–9.6)	7.4 (5.4–11.3)	8.3 (5.1–12.4)	<0.001
***Medications***				
β-blockers, n (%) (n = 244/40/107)	122 (50)	22 (55)	69 (64)	0.04
Ca-blocker, n (%) (n = 244/40/107)	80 (33)	11 (28)	43 (40)	0.26
ACEi/ARB, n (%) (n = 251/41/110)	149 (59)	18 (44)	58 (53)	0.13
Statins, n (%) (n = 251/41/110)	56 (22)	5 (12)	42 (38)	<0.001
Diuretics, n (%) (n = 249/41/109)	148 (59)	29 (71)	88 (81)	<0.001
***Pulmonary function***				
FVC (% predicted)	94 (83–112)	82 (59–118)	69 (51–78)	<0.001
FEV_1_ (% predicted)	98 (83–118)	63 (46–96)	73 (54–86)	<0.001
PEF (% predicted)	81 (49–118)	39 (23–85)	68 (40–97)	<0.001
FEV_1_/FVC (%)	85 (75–95)	65 (52–69)	88 (76–96)	<0.001
Pulse oxymetry (% saturation, n = 239/35/102)	98 (95–99)	98 (95–99)	98 (94–99)	0.45

Continuous variables are presented as median (10–90 percentile). Categorical variables are presented as number (n)/percentage (%). Abbreviations: Mean BP, mean blood pressure; GFR, glomerular filtration rate; Total BMD, Total bone mineral density; PEW, protein-energy wasting; SGA, Subjective global nutritional assessment; HDL, high-density lipoprotein; intact-PTH, intact parathyroid hormone; hsCRP, high-sensitivity C-reactive protein; IGF-1, insulin-like growth factor-1; IL-6, interleukin-6; TNF, tumor necrosis factor; ACEi, angiotensin-converting enzyme; ARB, angiotensin 2 receptor blocker; FVC, forced vital capacity; FEV_1_, forced expiratory volume in the first second; PEF, peak expiratory flow.

^a^Low physical activity or bed or wheelchair bound (according to questionnaire where patient reported one of four domains: 1) exercise frequently, 2) normal activity, 3) low activity, or 4) bedridden or wheelchair bound)

^b^ % HGS, Handgrip strength as percentage of values for health individuals. OLD; obstructive lung disorder FEV1/FVC <0.70, RLD; restrictive lung disorder, FEV1/FVC ≥0.70 and %FVC <80.

The clinical and biochemical characteristics of all investigated individuals placed in groups according to the separate GFR categories are shown in S1 Table. The levels of %FVC, %FEV_1_ and %PEF were lowest for GFR categories G3-5.

### Presence of obstructive and restrictive lung disorder

**Fig 1:**
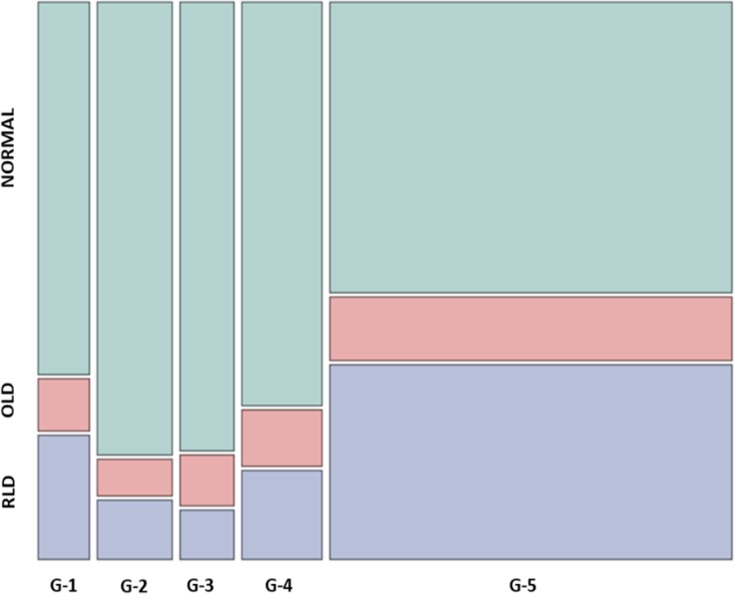
Prevalence of normal lung function, OLD and RLD in 404 individuals according to GFR categories 1–5. Chi square = 29.9, p<0.001. Abbreviations: OLD; obstructive lung disorder FEV1/FVC <0.70, RLD; restrictive lung disorder, FEV1/FVC ≥0.70 and %FVC <80. G-1 through G-5 are GFR categories: Normal (G1; n = 31; GFR >90mL/min/1.73 m2) or only mildly decreased (G2; n = 46) GFR, significant reductions of GFR, G3 (n = 33) and G4 (n = 49), and patients with kidney failure, G5 (n = 245; GFR<15mL/min/1.73 m2).

A large proportion (36%) of G5 patients had signs of restrictive lung disorder (RLD) while this was less common (14%) among G1-4 individuals; in contrast, the prevalence of obstructive lung disorder (OLD) was lower in G5 (11%) and G1-4 individuals (9%) (χ^2^ = 29.9, p<0.001) (**Fig 1**).

### Presence of PEW, inflammation and CVD in relation to lung dysfunction

Signs of PEW (SGA>1), inflammation or CVD were present in 176 (45%) of the 404 investigated individuals. S1 Fig shows the number of patients with one, two or three of these complications.

The prevalence of lung dysfunction, especially RLD, increased progressively in those exhibiting one, two or three of the complications, PEW, inflammation and CVD (χ^2^ = 72.9, p<0.001) (S2 Fig). In those with concomitant presence of all three components, 64% had RLD while lung function was normal in 79% of those without these complications (n = 223).

The highest tertile of Framingham’s score for CVD risk was associated with increased prevalence of lung dysfunction (χ^2^ = 10.9, p = 0.02) (S3 Fig).

### Multivariate analysis

Multivariate logistic regression analysis to determine independent predictors of lung dysfunction showed that RLD significantly associated with PEW, inflammation, albumin, CVD, and GFR category 3, while OLD associated with PEW, after adjustments for Framingham’s CVD risk score (thus taking into account age, sex, smoking, blood pressure, total cholesterol, HDL cholesterol and usage of anti-hypertensive medication), inflammation, serum albumin, and GFR category (Table 2). Results remained essentially the same when excluding patients with congestive heart failure or using prednisolone tablets or inhaler.

**Table 2:** Multivariate logistic regression analysis of factors associated with obstructive (OLD) and restrictive (RLD) lung dysfunction in 404 individuals with GFR categories G1-5.

	OLD		RLD	
	OR (95%CI)	p value	OR (95%CI)	p value
**FRS middle vs low tertile**	**0.72 (0.27–1.92)**	**0.52**	**0.52 (0.26–1.04)**	**0.07**
**FRS high vs low tertile**	**1.70 (0.68–4.30)**	**0.26**	**0.88 (0.44–1.75)**	**0.71**
**Cardiovascular disease, yes or no**	**1.36 (0.58–3.18)**	**0.47**	**3.10 (1.69–5.66)**	**<0.001**
**Malnutrition, SGA>1 vs SGA = 1**	**4.55 (1.81–11.4)**	**0.001**	**3.27 (1.59–6.69)**	**0.001**
**Albumin middle vs low tertile**	**0.62 (0.24–1.56)**	**0.31**	**0.58 (0.31–1.10)**	**0.10**
**Albumin high vs low tertile**	**0.88 (0.33–2.33)**	**0.80**	**0.44 (0.21–0.95)**	**0.04**
**hsCRP middle vs low tertile**	**1.62 (0.66–4.02)**	**0.30**	**2.88 (1.37–6.05)**	**0.005**
**hsCRP high vs low tertile**	**1.58 (0.59–4.24)**	**0.36**	**3.85 (1.80–8.27)**	**0.001**
**GFR category 3 vs GFR category 1,2**	**0.92 (0.19–4.43)**	**0.92**	**0.22 (0.05–0.94)**	**0.04**
**GFR category 4 vs GFR category 1,2**	**0.94 (0.23–3.87)**	**0.93**	**0.33 (0.10–1.07)**	**0.07**
**GFR category 5 vs GFR category 1,2**	**1.39 (0.44–4.43)**	**0.57**	**0.84 (0.36–1.96)**	**0.69**

Pseudo r^2^ = 0.16. Abbreviations: OR, odds ratio; FRS, Framingham CVD risk score; GFR, glomerular filtration rate; SGA, Subjective global nutritional assessment; hsCRP, high-sensitivity C-reactive protein. OLD; obstructive lung disorder FEV1/FVC <0.70, RLD; restrictive lung disorder, FEV1/FVC ≥0.70 and %FVC <80.

## Discussion

There are scarce reports about lung dysfunction and its implications in CKD individuals across various GFR categories. Whereas associations of nutritional and inflammatory status with pulmonary dysfunction have been reported in CKD stage 5 patients, non-dialyzed [25] as well as those undergoing hemodialysis or peritoneal dialysis [26], there are less such data for GFR categories G1-4. This study shows that restrictive lung disorder (RLD) is common in patients with CKD and associates with PEW, inflammation, CVD and degree of renal function impairment. In patients with concomitant presence of PEW, inflammation and CVD, as many as 64% had RLD. To the best of our knowledge, this is the first report on such an association using multivariate analysis with adjustments for adjustments for Framingham’s CVD risk score, PEW, inflammation, serum albumin, and GFR category.

These results suggest that RLD may be a consequence of pulmonary-cardio-renal interactions involving inflammation and PEW, two common features of advanced CKD. Thus, RLD appears to be an integral albeit scarcely explored complication in patients with advanced CKD. The association of RLD with the triad PEW, inflammation and CVD suggests that RLD may share similar pathophysiological mechanisms and pathways with the MIA syndrome [7].

There is also a scarcity of data on the prevalence of OLD and RLD across the CKD spectrum. In the present study, %FVC, %FEV_1_ and %PEF associated with GFR, and were lowest among those with GFR categories G3-5. RLD was more common in G5 (36%) than in G1-4 individuals (14%) whereas the prevalence of OLD was lower and similar in G5 (11%) and G1-4 individuals (9%). Two studies reported that the prevalence of chronic obstructive pulmonary disease (COPD) was 20%-30% among CKD patients [37, 38]; however, these patients had undergone surgical procedures that may represent a selection bias. However, a recent study reported frequencies of OLD (16%) and RLD (10%) in patients with CKD 1–4 [39] which were similar to those found in the present study.

In RLD, the pulmonary compliance of the thoracic wall and/or the lung itself is decreased. As the dynamic ventilatory capacity depends on the respiratory muscles, it could be influenced by muscle wasting in CKD patients with PEW and inflammation. Pro-inflammatory cytokines induce muscle protein breakdown [40] through ‘cytokine-driven’ pathways that increase protein catabolism [41]. A previous study showed that decreased respiratory muscle strength may lead to decreased vital capacity in CKD patients [42]. The self-reported physical activity level was low among those of our individuals with lung dysfunction. One may speculate that physical exercise to improve muscle strength could have a beneficial impact on the muscles of respiration [43].

While we did not investigate fluid status, it is well established that volume overload in patients with kidney failure is closely associated with restrictive and obstructive respiratory abnormalities [44], and in one study, 63% of investigated hemodialysis patients including even asymptomatic patients had signs of moderate to severe lung congestion [3]. Pulmonary congestion among hemodialysis patients is associated with a mixed restrictive-obstructive pattern on pulmonary function tests [45], which may be a consequence of liquid accumulation close to airways that leads to obstruction and dysfunction [46]. In addition to fluid overload, impaired pulmonary function may be the direct result of circulating uremic toxins or may indirectly result from other common complications in ESRD patients such as anemia, inflammation, malnutrition, electrolyte disorders, and acid-base imbalances [46]. Chronic inflammation enhances telomere shortening, which in turn has been shown to lead to senescence of lung alveolar and endothelial cells [47, 48]. Furthermore, endothelial dysfunction induced by systemic inflammation [49] may lead to pulmonary vascular filtration and lung tissue damage.

Findings of our study are in agreement with previous reports [50, 51] which showed association between restrictive lung function and CVD although the pathophysiological mechanism(s) underlying this relationship is still not well defined. Obstructive lung function manifested as COPD has been reported to increase systemic inflammation and elevate the risk of CVD [52] while restrictive lung function may be associated with an even greater increase in the risk of CVD compared to COPD [50, 51, 53]. A recent longitudinal study showed that reduced lung volume associates with inflammation [16]. Although it is unclear whether lung dysfunction leads to an inflammatory response, or vice versa, or if other mechanisms are involved, inflammation is known to be involved in the pathogenesis of atherosclerosis [54] which could help to explain the observed association between indices of reduced lung volume and clinical signs of CVD in the present study.

One intriguing finding in the present study, is that normal lung function was less common and thus that the prevalence of RLD and OLD was higher among G1 as compared to G2 and G3 individuals. One explanation could be that smoking was more common among G1 individuals (64.3%) than in G2 (59.1%) and G3 (55.2%) individuals—and only 23.5% of G5 patients were smokers. Most of those with RLD were treated with diuretics and other anti-hypertensive medications (Table 1), which may explain why there was no statistical difference of mean blood pressure among various groups. RLD has been reported to associate with proteinuria [55], and it is well established that persistent proteinuria is linked to atherosclerosis and CVD [56]. We found most of those with RLD had albuminuria (≥30mg/day).

Limitations of the present study include the relatively low number of participants and its observational cross-sectional design that does not permit conclusions concerning causality. Second, because of the selection bias with inclusion of only clinically stable CKD individuals with low prevalence (n = 24) of previously known lung disease, the findings may not necessarily be valid for other CKD patient populations. Third, we did not investigate several other factors, such as preceding time of uremia, fluid overload, extravascular lung water, pulmonary hypertension, subclinical pulmonary edema and interstitial fibrosis, that may promote restrictive lung impairment [2].

In summary, 36% of CKD patients with GFR <15 L/min/1.73m^2^ (GFR category G5), and 9–22% in other GFR categories, had signs of restrictive lung disorder (RLD). In addition to low GFR, restrictive lung function associated with CVD, PEW, and inflammation. RLD was found in 64% of those with concomitant presence of PEW, inflammation and CVD while 79% of those lacking these complications had normal lung function. This suggests that pulmonary-cardio-renal interactions may contribute to RLD in patients with advanced CKD in presence of inflammation and PEW. In contrast, obstructive lung disorder associated mainly with low BMI and was less common with similar prevalence (5–11%) across GFR categories. We conclude that RLD appears to be an integral albeit scarcely explored complication in patients with advanced CKD. Further studies on causes of this complication in CKD and its consequences in terms of clinical outcomes are warranted.

## Supporting information

S1 Table Clinical and biochemical characteristics and lung function data in 404 CKD patients with GFR categories G1–5. (PDF)

S1 Fig Prevalence of CVD, PEW (SGA>1) and inflammation in 399 individuals. (PDF)

S2 Fig Prevalence of RLD, OLD and normal lung function among 404 individuals in relation to number of concomitantly present comorbid conditions. (PDF)

S3 Fig Prevalence of RLD, OLD and normal lung function among 404 individuals in relation to tertiles of Framingham´s CVD risk score. (PDF)
