# Supplementary material for: Restrictive lung disorder is common in patients with kidney failure and associates with protein-energy wasting, inflammation and cardiovascular disease
Source: PLoS One. 2018 Apr 27;13(4):e0195585. doi: 10.1371/journal.pone.0195585 (PMC5922538; doi:10.1371/journal.pone.0195585)
Supplement: S1 Table — (PDF) [file pone.0195585.s001.pdf]

S1 Table. Clinical and biochemical characteristics and lung function data in 404 CKD patients with GFR categories G1 - 5.

	G 1 (n=31)	G 2 (n=46)	G 3 (n=33)	G 4 (n=49)	G 5 (n=245)	P value
Demography and clinical characteristics						
Age (years)	59 (38-69)	64 (49-77)	61 (30-74)	60 (37-75)	56(34-69)	<0.001
Males, n (%)	22 (71)	32 (70)	26 (79)	33 (67)	149 (61)	0.21
Diabetes mellitus, n (%)	2 (6.5)	1 (2.2)	8 (24.2)	20 (40.8)	83 (33.9)	<0.001
Cardiovascular disease, n (%)	3 (9.7)	2 (4.4)	9 (27.3)	13 (26.5)	84 (34.3)	<0.001
Smoker, n (%) (n=28/44/29/42/200)	18 (64.3)	26 (59.1)	16 (55.2)	21 (50)	47 (23.5)	<0.001
Mean BP (mmHg, n=31/46/33/49/244)	100 (87-117)	104 (89-123)	113 (87-133)	109 (93-123)	110 (92-130)	<0.001
eGFR (ml/min/1.73 m^2)	102 (93-130)	75 (64-89)	37 (31-55)	23 (15-29)	6.4 (3.7-11.1)	<0.001
Total BMD (g/cm^2, n=31/46/33/47/211)	1.2 (1.1-1.3)	1.2 (1.1-1.3)	1.2 (1.1-1.3)	1.2 (1.0-1.3)	1.1 (1.0-1.3)	0.02
Low physical activity [a], n (%) (n=31/45/29/45/225)	1 (3)	1 (2)	0 (0)	7 (15)	35 (15)	<0.001
Nutritional status						
PEW (SGA>1), n (%) (n=30/44/33/49/243)	1 (3.3)	1 (2.3)	0 (0)	8 (16)	53 (22)	<0.001
Body mass index (kg/m^2)	24.8 (20.3-31.1)	25.4 (20.4-31.2)	27.0 (22.2-31.7)	25.5 (21.0-33.1)	24.6 (19.7-30.8)	0.11
Lean body mass index (kg/m^2, n=31/44/31/47/237)	17.7 (14.4-20.3)	17.6 (14.7-21.3)	18.1 (14.9-21.3)	17.9 (14.8-21.0)	17.0 (13.6-19.9)	0.01
Fat body mass index (kg/m^2, n=31/44/31/47/237)	7.8 (4.5-12.3)	7.5 (4.6-10.7)	8.2 (4.7-12.5)	7.4 (4.8-12.3)	7.7 (4.2-11.9)	0.90
%HGS[b] (%, n=31/46/33/48/241)	100	100	97 (70-124)	100 (51-128)	88 (58-122)	<0.001
Markers of metabolism						
Creatinine (µmol/L)	70 (56-81)	90 (68-101)	155 (121-205)	250 (182-341)	691 (430-1060)	<0.001
Triglyceride (mmol/L, n=30/46/32/48/244)	1.1 (0.7-3.1)	1.0 (0.7-2.5)	1.6 (0.8-3.9)	1.9 (0.9-2.9)	1.8 (0.9-3.5)	<0.001
Total Cholesterol (mmol/L, n=30/46/32/49/245)	4.8 (3.6-6.5)	5.1 (4.1-6.6)	4.8 (3.9-6.0)	5.1 (3.7-7.0)	4.7 (3.2-6.7)	0.18
HDL Cholesterol (mmol/L, n=31/46/17/25/243)	1.5 (0.9-2.4)	1.4 (1.1-2.1)	1.1 (0.9-1.8)	1.2 (0.8-1.8)	1.2 (0.8-1.9)	<0.001

Calcium (mmol/L, n=30/46/33/49/241)	2.4 (2.1-2.4)	2.3 (2.2-2.4)	2.4 (2.3-2.6)	2.4 (2.2-2.6)	2.5 (2.1-2.8)	<0.001
Phosphate (mmol/L, n=30/46/33/49/240)	1.0 (0.7-1.2)	1.0 (0.8-1.2)	1.1 (0.7-1.5)	1.3 (1.0-1.6)	1.9 (1.3-2.7)	<0.001
Intact-PTH (ng/L, n=30/45/33/48/244)	34 (22-64)	37 (27-62)	84 (31-142)	105 (47-156)	211 (47-527)	<0.001
Circulating biomarkers						
Albumin (g/L)	39 (37-42)	39 (35-43)	38 (32-43)	37 (29-41)	34 (27-40)	<0.001
Hemoglobin (g/L)	142 (125-158)	146 (131-155)	132 (112-149)	124 (104-147)	107 (90-127)	<0.001
hsCRP (mg/L)	0.9 (0.4-8.1)	1.4 (0.4-5.5)	2.8 (0.5-10.0)	2.8 (0.6-12.3)	4.9 (0.7-28.4)	<0.001
IGF-1 (µg /mL, n=20/34/23/42/200)	132 (39-248)	111(70-159)	148(61-233)	136 (65-233)	186 (95-333)	<0.001
IL-6 (pg/mL, n=28/40/13/34/241)	1.7 (0.4-4.7)	2.1 (0.8-8.5)	3.3 (2.0-6.1)	2.5 (1.6-13.8)	6.1 (2.3-16.8)	<0.001
Fibrinogen (g/L, n=30/46/31/49/240)	3.1 (2.4-3.9)	2.8 (2.3-3.7)	3.2 (2.4-4.3)	3.9 (2.9-5.9)	4.7 (3.4-6.9)	<0.001
TNF (pg/mL, n=15/25/22/35/228)	3.9 (2.5-6.2)	3.7 (1.5-9.1)	6.6 (4.6-14.2)	8.3 (5.4-16.6)	11.4 (6.3-21.4)	<0.001
Leucocytes count (10^9/L)	5.7 (4.0-8.0)	6.0 (4.4-8.7)	6.1 (4.5-8.2)	7.5 (5.1-10.1)	7.6 (5.3-11.5)	<0.001
Medications						
β-blockers, n (%) (n=31/46/31/49/234)	6 (19)	9 (20)	15 (48)	30 (61)	153 (65)	<0.001
Ca-blocker, n (%) (n=31/46/31/49/234)	2 (6)	2 (4)	11 (35)	24 (49)	95 (41)	<0.001
ACEi/ARB, n (%) (n=31/46/31/49/245)	3 (10)	8 (17)	22 (71)	39 (80)	153 (62)	<0.001
Statins, n (%) (n=31/46/31/49/245)	7 (23)	4 (9)	9 (29)	13 (27)	70 (29)	0.04
Diuretics, n (%) (n=31/46/31/49/242)	0 (0)	5 (11)	18 (58)	37 (76)	205 (85)	<0.001
Pulmonary function						
FVC (% predicted)	88 (69-113)	97 (78-114)	103(75-117)	90 (58-109)	83 (59-105)	<0.001
FEV_1 (% predicted)	94 (78-117)	104 (81-123)	103 (69-118)	96 (49-113)	84 (57-106)	<0.001
PEF (% predicted)	87(49-119)	87 (56-126)	85(52-122)	75 (40-118)	69 (37-100)	<0.001
FEV_1/FVC (%)	86 (70-96)	85 (74-97)	84 (68-93)	84 (69-97)	85 (68-95)	0.52
Pulse oximetry (% saturation, n=30/45/31/46/224)	97 (94-99)	97 (95-99)	98 (95-99)	98 (96-99)	98 (95-99)	0.007
Normal lung function, n (%)	21 (68)	38 (83)	27 (82)	36 (74)	130 (53)	

OLD, n (%)	3 (10)	3 (6)	3 (9)	5 (10)	28 (11)	<0.001
RLD, n (%)	7 (22)	5 (11)	3 (9)	8 (16)	87 (36)	
Albuminuria (mg/24 hours) (n=26/44/26/40/145)	0 (0-7)	0 (0-51)	308 (4-1469)	188 (12-1389)	1458 (92-6196)	<0.001
Framingham CVD score (%)	14.1 (2.3-47.8)	20.2 (7.2-52.2)	24.4 (2.8-75.3)	20.2 (5.9-86.1)	21.8 (4.4-55.6)	0.12

Continuous variables are presented as median (10 – 90 percentile). Categorical variables are presented as number (n)/percentage (%). Abbreviations: Mean BP, mean blood pressure; GFR, glomerular filtration rate; Total BMD, Total bone mineral density; SGA, Subjective global nutritional assessment; HDL, high-density lipoprotein; intact-PTH, intact parathyroid hormone; hsCRP, high-sensitivity C-reactive protein; IGF-1, insulin-like growth factor-1; IL-6, interleukin-6; TNF, tumor necrosis factor; ACEi, angiotensin-converting enzyme; ARB, angiotensin 2 receptor blocker; FVC, forced vital capacity; FEV_1, forced expiratory volume in the first second; PEF, peak expiratory flow. [a]Low physical activity or bed or wheelchair bound (according to questionnaire where patient reported one of four domains: 1) exercise frequently, 2) normal activity, 3) low activity, or 4) bedridden or wheelchair bound); [b] % HGS, Handgrip strength as percentage of values for CKD 1 and 2. OLD; obstructive lung disorder FEV1/FVC <0.70, RLD; restrictive lung disorder, FEV1/FVC ≥0.70 and %FVC <80.
