# Supplementary material for: Restrictive lung disorder is common in patients with kidney failure and associates with protein-energy wasting, inflammation and cardiovascular disease
Source: PLoS One. 2018 Apr 27;13(4):e0195585. doi: 10.1371/journal.pone.0195585 (PMC5922538; doi:10.1371/journal.pone.0195585)

S1 Fig. Prevalence of CVD, PEW (SGA>1) and inflammation in 399 individuals.
The figure shows whether one, two or three components of these conditions were present. In 223 individuals, there were no signs of CVD, PEW and inflammation. SGA data (n=5) was missing.

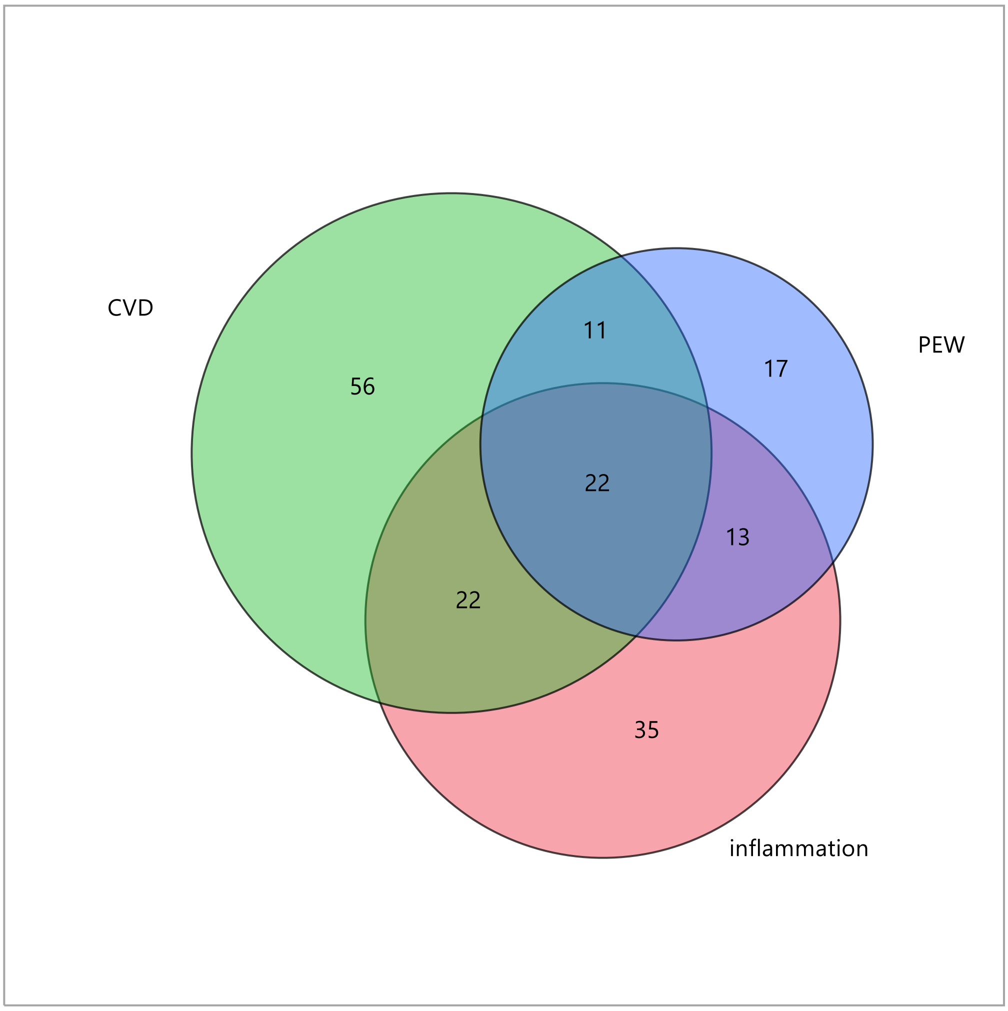

Supplement: S1 Fig — (PDF) [file pone.0195585.s002.pdf]
