# Supplementary material for: Restrictive lung disorder is common in patients with kidney failure and associates with protein-energy wasting, inflammation and cardiovascular disease
Source: PLoS One. 2018 Apr 27;13(4):e0195585. doi: 10.1371/journal.pone.0195585 (PMC5922538; doi:10.1371/journal.pone.0195585)
Supplement: S2 Fig — (PDF) [file pone.0195585.s003.pdf]

S2 Fig. Prevalence of RLD, OLD and normal lung function among 404 individuals to number of concomitantly present comorbid conditions, i.e., PEW, inflammation and CVD.
Chi square=75.0, p<0.001.

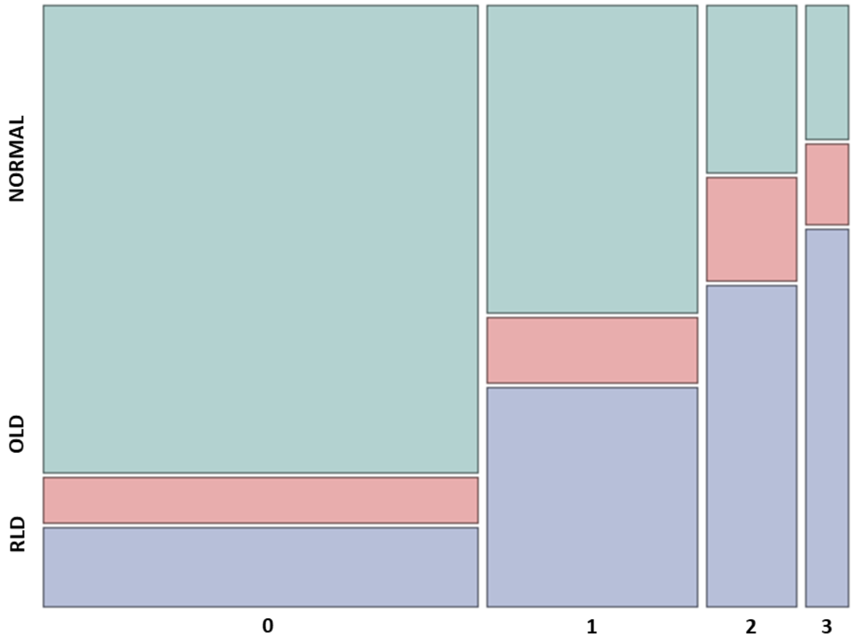

Abbreviations: OLD; obstructive lung disorder FEV1/FVC <0.70, RLD; restrictive lung disorder, FEV1/FVC ≥0.70 and %FVC <80.
